# Risk factors for healing failure after arthroscopic rotator cuff repair in small to medium-sized tears: a retrospective cohort study

**DOI:** 10.3389/fsurg.2024.1456540

**Published:** 2024-11-11

**Authors:** Guangying Wang, Changli Liu, Jiansong Wang, Haoran Li, Guosheng Yu

**Affiliations:** ^1^ Graduate School, Hebei University of Chinese Medicine, Shijiazhuang, China; ^2^ Department of Sports Medicine, The Cangzhou Hospital of Integrated TCM-WM Hebei, Cangzhou, China

**Keywords:** rotator cuff tears, healing failure, risk factors, acromiohumeral distance, arthroscopic surgery, fatty infiltration

## Abstract

**Objectives:**

To identify risk factors for tendon healing failure following arthroscopic rotator cuff repair (ARCR) in patients with small to medium-sized rotator cuff tears (RCTs).

**Methods:**

A retrospective study was conducted on 320 patients with RCTs who underwent arthroscopic repair between June 2018 and June 2021. All patients had at least 2 years of postoperative follow-up, with MRI scans at the final assessment. Based on MRI results, patients were categorized into the healing success group (Group A: types I–III) or the healing failure group (Group B: types IV–V). Variables associated with rotator cuff healing, including patient characteristics, baseline symptoms, imaging data, and surgery-related factors, were analyzed using univariate and multivariate logistic regression.

**Results:**

Healing failure occurred in 54 of the 320 patients (16.9%). Functional status improved significantly across all patients (*P* < 0.05), irrespective of healing outcomes. Multifactorial analysis identified smoking (OR = 1.931, *P* = 0.028), diabetes (OR = 3.517, *P* = 0.038), lower bone mineral density (BMD) (OR = 1.551, *P* = 0.018), higher fatty infiltration (FI) (OR = 4.025, *P* = 0.009), and smaller acromiohumeral distance (AHD) (OR = 2.546, *P* = 0.006) as independent risk factors for healing failure.

**Conclusions:**

Smoking, diabetes, lower BMD, higher FI, and smaller AHD are independent risk factors for healing failure following ARCR.

## 1 Introduction

Rotator cuff tears (RCTs) are a leading cause of shoulder pain and significantly reduce patients’ quality of life, making them a growing global health concern (1). The prevalence of RCTs is approximately 13% in individuals over 50, increasing to 25% in those over 60 and up to 50% in those over 80 years old (2). As shoulder arthroscopy becomes more advanced, the arthroscopic rotator cuff repair (ARCR) technique is increasingly used in patients with RCTs, particularly when conservative treatments have failed (3), yielding favorable outcomes. Achieving anatomic integrity of the repaired tendon is the primary goal of ARCR. However, healing failure and postoperative retears remain significant complications (4), with reported incidence rates between 13% and 84% (5).

Small to medium-sized RCTs, defined by Cofield et al. (6) as tears involving less than 3 cm of the tendon, generally have favorable clinical and anatomic outcomes after surgical repair. However, some patients still experience healing failure and postoperative pain. While multivariate analyses have been done on large to massive tears, limited research exists for smaller tears (7, 8). Therefore, this study aims to evaluate ARCR outcomes for small to medium-sized tears and identify the risk factors that affect their healing.

## 2 Materials and methods

### 2.1 Patient selection

Clinical data of patients with RCTs who underwent ARCR between June 2018 and June 2021 at the Cangzhou Hospital of Integrated TCM-WM Hebei were analyzed. The inclusion criteria were as follows: (1) diagnosis of full-thickness RCTs treated with ARCR; (2) availability of complete clinical data; and 3. follow-up of at least 2 years, including MRI examination at the final follow-up. The exclusion criteria were as follows: (1) partial-thickness RCTs; (2) isolated subscapularis tendon tears; (3) large to massive tears per the DeOrio et al. classification (9), and (4) history of previous shoulder surgery. Out of the 653 patients initially considered, 320 patients were ultimately included in the analysis. The reasons for this reduction primarily involved non-compliance with initial inclusion criteria, withdrawal of informed consent, and loss to follow-up. A total of 320 patients with small to medium-sized full-thickness RCTs met the inclusion criteria, comprising 150 males (46.9%) and 170 females (53.1%). Of these, 106 patients underwent the single-row suture technique, and 214 underwent with double-row suture technique. Based on the most recent MRI results, patients were categorized into two groups: the healing success group (type I–III) and the healing failure group (type IV–V). The study was conducted with approval from the ethics committee of our hospital, and written informed consent was obtained from all participants, in accordance with the Declaration of Helsinki.

### 2.2 Clinical variables

The demographic variables analyzed in this study included age, sex, BMI, smoking, drinking, heart disease, hypertension, and diabetes (Table 1). Clinical variables assessed included symptom duration, history of steroid injections, shoulder stiffness, dominant hand involvement, range of motion (ROM), bone mineral density (BMD), fatty infiltration (FI), tear size, cuff retraction, and acromiohumeral distance (AHD). Additionally, intraoperative variables such as acromioplasty, biceps surgery, and the type of repair technique were recorded and included in the analysis (Table 2).

**Table 1:** The main demographic variables between the two groups of patients.

Characteristics	Group A (*n* = 266)	Group B (*n* = 54)	*P* value
Age, years	63.8 ± 6.0	62.1 ± 6.5	0.076
Sex, *n* (%)			0.842
Male	121 (45.4%)	29 (53.7%)	
Female	145 (54.5%)	25 (46.3%)	
BMI, kg/m^2^			0.691
≤27	199 (74.8%)	39 (72.2%)	
>27	67 (25.2%)	15 (27.8%)	
Smoking			0.029*
Yes	105 (39.5%)	30 (55.6%)	
No	161 (60.5%)	24 (44.4%)	
Drinking			0.662
Yes	112 (42.1%)	21 (38.9%)	
No	154 (57.9%)	33 (61.1%)	
Heart disease			0.396
Yes	126 (47.4%)	29 (53.7%)	
No	140 (52.6%)	25 (46.3%)	
Hypertension			0.458
Yes	138 (51.9%)	31 (57.4%)	
No	128 (48.1%)	23 (42.5%)	
Diabetes			0.017*
Yes	46 (17.3%)	17 (31.5%)	
No	220(82.7%)	37(68.5%)	

BMI, body mass index.

* *P* < 0.05.

**Table 2:** Comparison of the related risk factors between the two groups of patients treated with ARCR.

Characteristics	Group A (*n* = 266)	Group B (*n* = 54)	*P* value
Symptom duration, mo	15.5 ± 4.3	16.4 ± 5.9	0.272
Steroid injection history			0.765
Yes	54 (20.3%)	10 (18.5%)	
No	212 (79.7%)	44 (81.5%)	
Shoulder stiffness			0.560
Yes	64 (24.1%)	11 (20.4%)	
No	202 (75.9%)	43 (79.6%)	
Dominant hand			0.817
Yes	203 (76.3%)	42 (77.8%)	
No	63 (23.7%)	12 (22.2%)	
ROM			
Forward flexion, deg	151.9 ± 15.3	148.9 ± 21.8	0.447
External rotation, deg	62.4 ± 16.6	59.2 ± 17.4	0.199
Internal rotation, deg	9.4 ± 3.6	8.8 ± 3.1	0.242
BMD	−1.11 ± 1.34	−1.61 ± 1.42	0.013
FI			<0.001**
<2 Grade	205 (77.1%)	29 (53.7%)	
≥2 Grade	61 (22.9%)	25 (46.3%)	
Tear size			<0.001**
≤2 cm	236 (88.7%)	38 (70.4%)	
>2 cm	30 (11.3%)	16 (29.6%)	
Retraction of the cuff			0.016*
≤2 cm	224 (84.2%)	38 (70.4%)	
>2 cm	42 (15.8%)	16 (29.6%)	
AHD, cm	8.47 ± 1.49	7.15 ± 2.23	<0.001**
Acromioplasty			0.484
Yes	190 (71.4%)	36 (66.7%)	
No	76 (28.6%)	18 (33.3%)	
Biceps surgery			0.035*
Yes	97 (36.5%)	28 (51.9%)	
No	169 (63.5%)	26 (48.1%)	
Repair technique			0.024*
Single-row	81 (30.5%)	25 (46.3%)	
Double-row	185(69.5%)	29(53.7%)	

ROM, range of motion; BMD, bone mineral density; FI, fatty infiltration; AHD, acromiohumeral distance.

* *P* < 0.05, **P = 0.000.

### 2.3 Imaging and clinical evaluation

**Figure 1:**
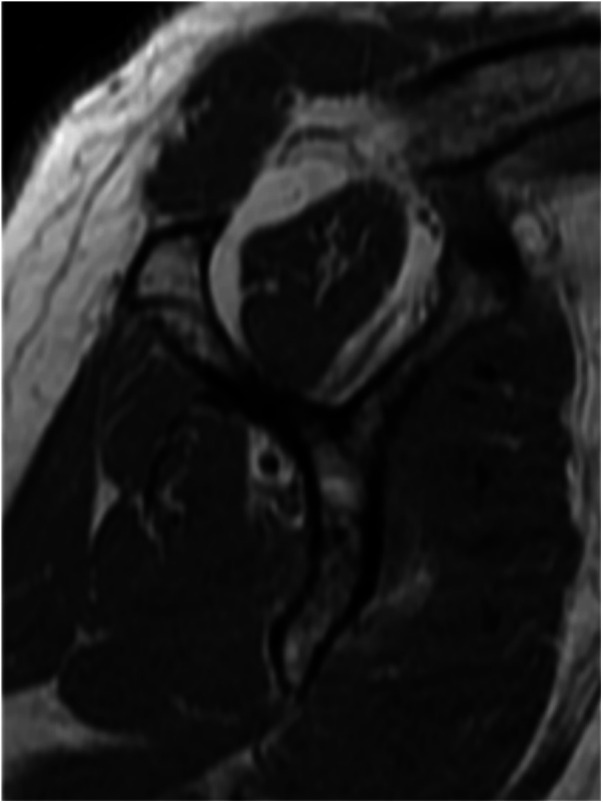
The patient's rotator cuff muscle fatty infiltration was assessed in the oblique sagittal scapular Y position on MRI T1-weighted images. This case was grade 2 fatty infiltration.

**Figure 2:**
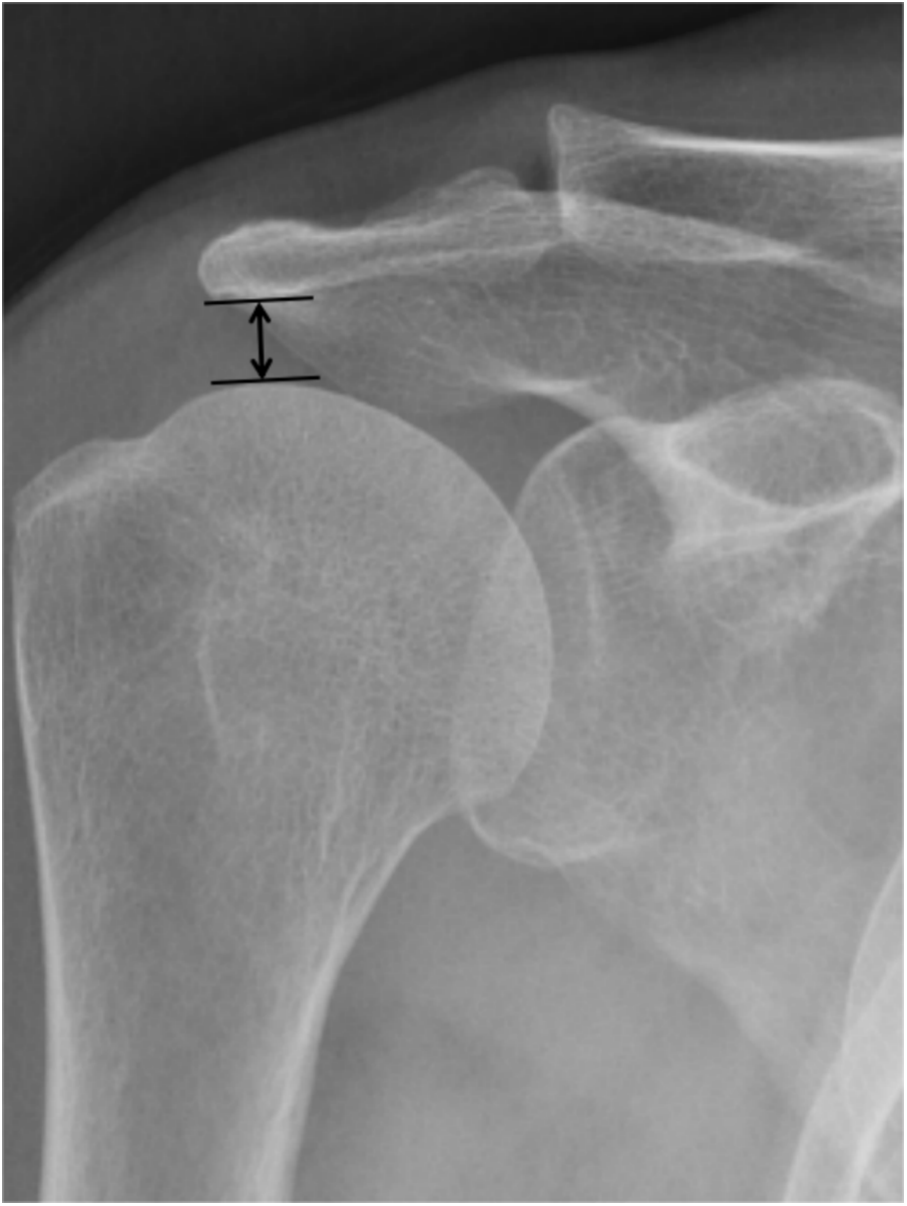
The acromiohumeral distance was defined as the shortest distance between the dense cortical bone on the inferior side of the acromion and the subchondral cortex on the superior side of the humeral head (arrow). This case was 7.37 mm.

All patients underwent preoperative MRI to confirm the diagnosis and assess key parameters like tear size, cuff retraction, and the degree of FI. The tear size was calculated as the linear distance between the anterior and posterior margins on oblique sagittal T2-weighted images (T2WI). The extent of retraction was measured as the straight-line distance between the medial margin of the supraspinatus footprint and the medial margin of the retracted cuff on oblique coronal T2WI (10). The degree of FI of the rotator cuff muscle was evaluated using T1-weighted MRI in the oblique sagittal scapular Y position, following the classification criteria of Goutallier et al. (11) and the diagnostic method of Fuchs et al. (12) (Figure 1). FI was categorized into 5 grades, where higher grades indicated more extensive FI. Grade 0 represented no FI, grades 1 and 2 reflected mild FI with up to 50% normal muscle tissue, grade 3 indicated 50% FI, and grade 4 represented FI exceeding 50% of normal muscle tissue. According to Golding, (13) the AHD was measured as the distance between the inferior border of the acromion and the superior border of the humerus on a radiograph of the shoulder joint (Figure 2). BMD was assessed using dual-energy x-ray absorptiometry within one year prior to surgery. The lowest T-score from either the proximal femur or the lumbar spine was used for analysis.

All patients underwent MRI evaluations at least 2 years post-surgery to assess anatomical outcomes. Tendon healing success was determined using Sugaya et al.'s (14) classification on T2WI in both coronal and sagittal oblique views. This classification consists of 5 types: type I, where the tendon appears uniformly low-signal and sufficiently thick; type II, indicating a locally high-intensity signal but sufficient thickness; type III, showing tendon continuity despite insufficient tendon thickness; type IV, signifying a small discontinuity in more than one piece, indicating a minor retear; and type V, representing a severe retear. Healing failure was defined as types IV or V. All imaging evaluations were conducted by an experienced musculoskeletal radiologist.

ROM measurements of the affected shoulder, including forward flexion, external rotation, and internal rotation, were performed using a goniometer with the elbow bent at 90°. Pain was assessed with the Visual Analogue Scale (VAS), where 0 indicates no pain and 10 signifies the most severe pain. Pre- and postoperative functional outcomes were measured using the Constant-Murley score and the American Shoulder and Elbow Surgeons (ASES) score, with higher scores reflecting better shoulder function (15).

### 2.4 Surgical procedures and rehabilitation

All patients were treated by the same medical team and operated on by a senior surgeon. Surgeries were performed under general anesthesia with the patient in the beach chair position. For patients with a stiff shoulder, capsular release was performed following shoulder manipulation. In the glenohumeral joint, synovectomy and debridement of partially torn rotator cuff tendons were carried out. If the long head of the biceps tendon was intact and of good quality, it was preserved. Conversely, it was detached from the glenoid labrum and fixed in the intertubercular sulcus. The transverse humeral ligament was also severed to reduce tension. If the patient had a type II or type III acromion, acromioplasty was performed, polishing the acromion to type I. After the tear size, tendon quality, and patient's functional demands were evaluated, it was decided to use the single-row or double-row suture bridge technique. For patients with tears smaller than 2 cm, good tendon tissue quality, and no need for high-level functional demands, single-row repair is used. Conversely, double-row repair is applied. After placing the anchor according to the selected technique, the loaded suture was passed through the tendon using a flexible suture piercer. After securing the medial row sutures, an anchor without sutures is placed at the lateral aspect of the greater tuberosity. The sutures are then tensioned and pressed over the footprint to ensure proper reattachment of the tendon (16).

After surgery, the same rehabilitation team supervised the patients’ recovery exercises, tailoring the rehabilitation plan according to the severity of the rotator cuff injury. The rehabilitation process was divided into three stages. In the first stage, from the first day to 6 weeks after surgery, the affected shoulder was immobilized using an abduction brace. Passive range of motion exercises may begin at 2 weeks post-operation, but no active movement is allowed. In the second stage, from 6 weeks to 3 months after surgery, the goal during this phase is to restore the active range of motion while ensuring the protection of the surgical repair. Begin active-assisted and active exercises within a safe range. In the third stage, from 3 months to 6months after surgery, functional activities and sport-specific exercises are introduced gradually, based on the patient's tolerance. Strengthen the rotator cuff and surrounding muscles to restore full shoulder function.

### 2.5 Statistical analysis

SPSS 23.0 (IBM, Armonk, NY, USA) statistical software was utilized for data analysis, with a significance level set as *α* = 0.05. Measurement data were compared between the two groups using the independent samples *t*-test or nonparametric test, depending on normal distribution and variance homogeneity. Categorical data were analyzed with the chi-square test. Multivariate logistic regression analysis was performed on factors found to be statistically significant in the univariate analysis, identifying potential predictors of healing failure.

## 3 Results

A total of 320 patients were included in the study, with all surgeries successfully completed. No complications such as infection, wound issues, deltoid problems, or neurovascular injury were observed during follow-up. Based on MRI results, 266 patients had successful rotator cuff healing, while 54 patients experienced healing failure post-surgery, forming the two study groups. There were no significant differences in age, sex, BMI, drinking, heart disease, or hypertension between the two groups (*P* > 0.05) (Table 1).

In the univariate analysis, rotator cuff healing failure was significantly higher among smokers and diabetic patients (all *P* < 0.05). Additionally, lower BMD (*P* = 0.013), higher FI (*P* < 0.001), larger tear and retraction size (all *P* < 0.05), smaller AHD (*P* < 0.001), biceps surgery (*P* = 0.035), and different repair techniques (*P* = 0.024) were significantly associated with healing failure (Table 2).

With rotator cuff healing failure as the dependent variable, logistic regression analysis was performed with the variables that showed statistically significant differences in univariate analysis (Table 2). The analysis revealed that smoking (OR = 1.931, *P* = 0.028), diabetes (OR = 3.517, *P* = 0.038), lower BMD (OR = 1.551, *P* = 0.018), higher FI (OR = 4.025, *P* = 0.009), and smaller AHD (OR = 2.546, *P* = 0.006) were positively correlated with rotator cuff healing failure after ARCR. These factors were identified as independent risk factors for postoperative healing failure (Table 3).

**Table 3:** Multivariate logistic regression analysis for the risk factors for healing failure after ARCR.

Risk factor	OR (95% CI)	*P* value
Smoking	1.931 (1.448–4.623)	0.028*
Diabetes	3.517 (1.571–12.631)	0.038*
BMD	1.551 (0.402–3.768)	0.018*
FI	4.025 (2.609–10.385)	0.009*
AHD	2.546 (1.311–3.859)	0.006*

BMD, bone mineral density; FI, fatty infiltration; AHD, acromiohumeral distance.

* *P* < 0.05.

All patients achieved significant improvements in functional outcomes, including VAS, ASES, and Constant-Murley scores, at the last follow-up (all *P* < 0.001) (Table 4).

**Table 4:** Functional outcomes after arthroscopic repair in small to medium-sized RCTs.

Characteristics	Preoperatively	Final follow-up	*P* value
ROM			
Forward flexion, deg	150.9 ± 18.3	165.2 ± 10.12	<0.001**
External rotation, deg	61.3 ± 14.6	71.1 ± 12.4	<0.001**
Internal rotation, deg	9.3 ± 3.6	10.4 ± 2.8	0.213
VAS pain score	7.2 ± 1.7	1.1 ± 0.9	<0.001**
ASES score	52.6 ± 18.2	85.3 ± 13.3	<0.001**
Constant-Murley score	48.8 ± 15.1	78.6 ± 12.3	<0.001**

ROM, range of motion; VAS, visual analogue scale; ASES, American shoulder and elbow surgeons.

** *P* = 0.000.

## 4 Discussion

ARCR is a widely used minimally invasive procedure for the treatment of RCTs, providing pain relief, restoring function, and improving patients’ quality of life. However, despite technological advancements, rotator cuff healing failure remains a significant issue over the past few decades (17). Therefore, this study identifies potential risk factors for rotator cuff healing failure by measuring the incidence of rotator cuff healing failure in a retrospective study of RCTs patients treated with ARCR. In this retrospective study, the failure rate was 16.9%, aligning with previous studies (2, 18, 19). Various previous studies have identified prognostic factors that influence cuff healing after rotator cuff repair. Oh JH et al. found that age, retraction, FI, BMD, and work activity level were independent prognostic factors of healing failure in full-thickness RCTs (20, 21). Another study focusing on massive RCTs identified FI as an independent risk factor for structural integrity (7). For small to medium-sized tears, research has highlighted somking, FI, age, and tear size as significant prognostic factors (22, 23). Additionally, several authors have noted that preoperative AHD affects rotator cuff integrity post-ARCR (24, 25). This current study incorporated previous findings with a longer follow-up period.

Several previous studies suggest that metabolic diseases may interfere with rotator cuff healing due to their effects on bone, tendon quality, and circulation, all of which are crucial for tendon-bone healing (26, 27). It has been reported that metabolic conditions like diabetes are associated with higher risks of healing failure (28, 29). Diabetes has been linked to an increased risk of rotator cuff rupture and poorer functional outcomes post-arthroscopic tendon repair (30). Egemen et al. (31) observed the effects of diabetes on tendon injuries in animals and found delayed tissue regeneration and functional recovery, attributing these issues to a hyperglycemic environment that weakens tendon biomechanical properties and causes histopathological changes that ultimately lead to inadequate tendon repair, maintenance, and remodeling (32). A recent clinical study also confirms diabetes as a factor in postoperative healing failure (33). Consistently with previous data, our findings suggest that diabetes is a significant independent risk factor for healing in small to medium-sized RCTs.

Smoking as a risk factor for tendon healing is a subject of debate. Multiple studies have examined its potential negative effects on rotator cuff healing (34–36). Lundgreen et al. (34) found that tendon samples from smokers showed more advanced degeneration, increased apoptotic cells, decreased tenocyte density, and heightened proliferative activity compared to non-smokers. Similarly, Park et al. (35) reported that smoking deteriorates tendon quality, which may impair rotator cuff healing and increase the chances of RCTs. However, the effect of smoking on outcomes after rotator cuff repair remains an unanswered question. In contrast, McElvany et al. (36) reported in their meta-analysis that studies with a higher proportion of smokers showed more favorable results. In this study, although smoking was significantly associated with rotator cuff healing failure (OR = 1.931, *P* = 0.028), we did not observe significant differences in functional outcomes between these two groups. Additionally, smoking cessation has been shown to reduce postoperative complications in surgical patients (37). Therefore, attention should be given to smokers undergoing rotator cuff repair, particularly heavy smokers, and our surgeons strongly advise quitting smoking both before and after the procedure.

FI after RCTs is a complex pathophysiological phenomenon that significantly affects patient outcomes and clinical decision-making. Prolonged unrepaired rotator cuff damage increases the risk of FI, which can significantly reduce the success of surgical treatment. Greater preoperative FI is associated with a higher recurrence rate post-repair (11, 23, 38, 39). Longo et al. (40) reported that patients with moderate or significant FI (grades 2–4) had a significantly higher retear rate than those with minimal or no FI (grades 0–1). Our findings revealed that grade 2 or greater FI serves as a critical threshold influencing structural integrity after rotator cuff repair, with a notably higher rate of retear when FI equals or exceeds grade 2. FI (OR = 4.025, *P* = 0.009) was associated with an increased risk of postoperative retear. The irreversibility of FI after surgery may explain why patients with preoperative rotator cuff FI exhibit poorer shoulder function compared to those without FI. Therefore, we should focus more on the degree of FI in patients with RCTs during clinical practice and routinely assess and grade FI. Regarding the clinical evaluation of muscle FI, MRI remains the gold standard for evaluating rotator cuff FI. While the semi-quantitative Goutallier grading method is commonly used, new imaging techniques, such as magnetic resonance spectroscopy and water-fat separation, offer improved accuracy in FI evaluation and present distinct advantages (41, 42).

A lower BMD is a well-established risk factor for rotator cuff healing failure, as it implies reduced bone strength and structural integrity (20). Patients with lower BMD are likely to have more severe osteoporosis, which weakens the humeral greater tuberosity. We believe that this weakened bone structure contributes to anchor loosening and biological healing disorders, leading to rotator cuff healing failure after surgical repair. This finding was supported by biomechanical studies by Yakacki et al. (43) and Tingart et al. (44), who linked the quality of the humeral head bone to the pullout strength of suture anchors. Additionally, another biological mechanism may explain the relationship between osteoporosis and tendon-bone healing. Osteoporosis is characterized by excessive osteoclast activity (45), which may interfere with bone growth at the tendon-bone junction, resulting in weakness in this area. Therefore, the impact of BMD on rotator cuff healing failure is likely the result of multiple factors. In our study, all patients were prescribed long-term and regular anti-osteoporotic medications following surgery.

The AHD, defined as the shortest distance between the inferior border of the acromion and the head of the humerus, is a significant factor in determining rotator cuff function (25). An AHD of less than 6 mm, as measured on standard radiographs, is typically associated with larger RCTs (46). The relationship between RCTs and AHD was such that the incidence of tears increased as AHD decreased (24, 47, 48). Additionally, Saupe et al. demonstrated a negative correlation between tear size, the degree of FI in the rotator cuff muscles, and AHD, concluding that FI of the infraspinatus muscle had the most pronounced impact on AHD (49). When the infraspinatus muscle loses its ability to lower the humeral head, upward movement occurs due to the torn supraspinatus, which no longer functions as a placeholder (50). When the AHD is reduced, it places increased upward forces on the rotator cuff, particularly the supraspinatus tendon, within the limited space of the subacromial space, leading to more severe impingement (51). Furthermore, there is a reciprocal relationship between RCTs and abnormal AHD. A reduced AHD can accelerate the progression of RCTs, while the instability of the glenohumeral joint following RCTs causes further upwards migration of the humeral head, exacerbating the narrowing of AHD (52, 53). However, the impact of the AHD remains debated, with some studies finding no correlation between AHD and functional outcomes (54). Kim et al. (55) suggested that an increase in the size of sagittal retear and a decrease in AHD are significant imaging parameters for predicting retear rates. Nové-Josserand et al. (56) reported complete AHD narrowing following muscle fat degeneration in the supraspinatus muscle. Our study observed normal AHD in patients with stage 2 or 3 FI of the supraspinatus muscle, indicating a need for further investigation. Thus, the relationship between AHD and rotator cuff healing failure requires more clarification. While MRI provides moderate evidence for AHD measurement reliability (57), x-ray remains a practical and cost-effective alternative, offering similar sensitivity and predictive value for RCTs (58).

It must be noted that these interpretations are based on general understanding and assumptions. The exact mechanism by which these risk factors lead to healing failure after ARCR in patients with RCTs requires further study and may involve a combination of mechanical, inflammatory, neurological, and psychological factors. Future studies, including prospective and experimental research, could provide more insights into these underlying mechanisms and help guide strategies for preventing and managing postoperative nonunion in this patient group.

This study has several limitations. First, due to the retrospective study design, some patients who underwent rotator cuff repair surgery at our institution withdrew from the study, with only 320 patients enrolled out of 652. Second, the tear size, degree of FI, retraction distance, and healing status were measured and evaluated via MRI. Due to differences in MRI machines and scanning parameters, artifacts from implants, and variations in scanning planes and slice thickness, some measurement discrepancies may occur. These factors could further affect the study's results. Third, two different surgical techniques, single-row and double-row, were used in this study. The differences in biomechanical stability and healing potential between them may have led to some variability in the outcomes. Finally, being a single-center study with a limited sample size and short follow-up period, long-term and multistage follow-up is necessary to further validate our findings.

## 5 Conclusions

Smoking, diabetes, lower BMD, higher FI, and smaller AHD were identified as independent risk factors for rotator cuff healing failure following ARCR.

## Data Availability

The original contributions presented in the study are included in the article/Supplementary Material, further inquiries can be directed to the corresponding author.
